# The South East Asian Federation of Organizations for Medical Physics (SEAFOMP): Its history and role in the ASEAN countries

**DOI:** 10.2349/biij.4.2.21

**Published:** 2008-04-01

**Authors:** KH Ng, JHD Wong

**Affiliations:** 1 Department of Biomedical Imaging, University of Malaya, Kuala Lumpur, Malaysia; 2 Medical Physics Unit, University of Malaya Medical Centre, Kuala Lumpur, Malaysia

**Keywords:** Southeast Asia, SEAFOMP, history, medical physics, organization

## Abstract

Informal discussion started in 1996 and the South East Asian Federation of Organizations for Medical Physics (SEAFOMP) was officially accepted as a regional chapter of the IOMP at the Chicago World Congress in 2000 with five member countries, namely Indonesia, Malaysia, Philippines, Singapore and Thailand. Professor Kwan-Hoong Ng served as the founding president until 2006. Brunei (2002) and Vietnam (2005) joined subsequently. We are very grateful to the founding members of SEAFOMP: Anchali Krisanachinda, Kwan-Hoong Ng, Agnette Peralta, Ratana Pirabul, Djarwani S Soejoko and Toh-Jui Wong.

The objectives of SEAFOMP are to promote (i) co-operation and communication between medical physics organizations in the region; (ii) medical physics and related activities in the region; (iii) the advancement in status and standard of practice of the medical physics profession; (iv) to organize and/or sponsor international and regional conferences, meetings or courses; (v) to collaborate or affiliate with other scientific organizations.

SEAFOMP has been organizing a series of congresses to promote scientific exchange and mutual support. The South East Asian Congress of Medical Physics (SEACOMP) series was held respectively in Kuala Lumpur (2001), Bangkok (2003), Kuala Lumpur (2004) and Jakarta (2006). The respective congress themes indicated the emphasis and status of development. The number of participants (countries in parentheses) was encouraging: 110 (17), 150 (16), 220 (23) and 126 (7).

In honour of the late Professor John Cameron, an eponymous lecture was established. The inaugural John Cameron Lecture was delivered by Professor Willi Kalender in 2004. His lecture was titled “Recent Developments in Volume CT Scanning”.

## SOUTH-EAST ASIA AND ASEAN

**Figure 1:**
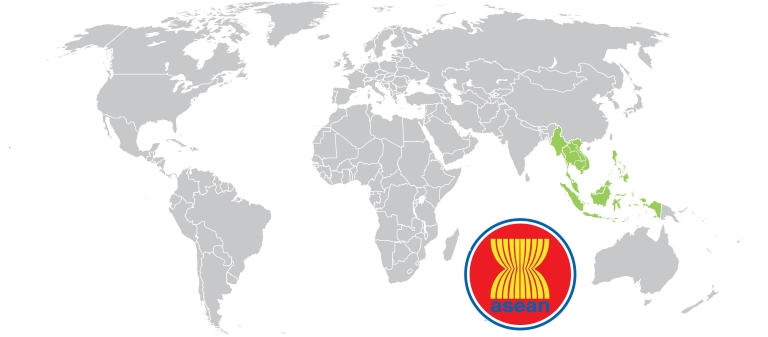
Map of South East Asia.

The Association of Southeast Asian Nations, commonly referred to as ASEAN, is an organization comprising of 10 countries located in Southeast Asia (Figure 1). The organization was formed on 8 August 1967 by its five original member countries, i.e. Indonesia, Malaysia, Philippines, Singapore and Thailand. Over the years, the organization grew when Brunei Darussalam joined in as the sixth member on 8 January 1984, Vietnam on 28 July 1995, Laos and Myanmar on 23 July 1997 and Cambodia on 30 April 1999. Its objectives include the acceleration of economic growth, social progress and cultural development among its members, as well as to promote regional peace [1].

## THE BIRTH OF SEAFOMP

**Figure 2:**
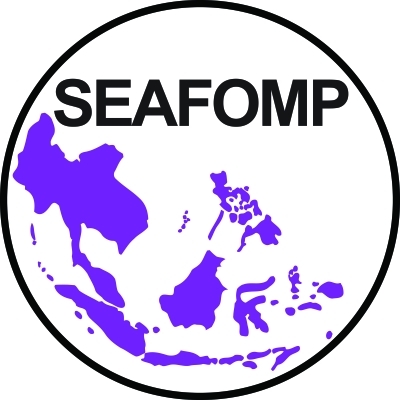
SEAFOMP logo.

**Figure 3:**
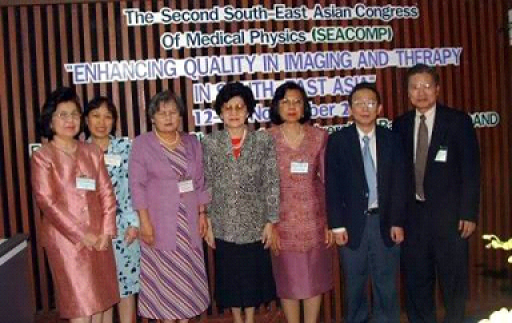
SEAFOMP founding members posing with a plenary speaker (4th from the left).

The spirit of ASEAN is resounded in SEAFOMP. The idea of setting up an organization for South-east Asian medical physics societies was first mooted in 1996. During the International Organization of Medical Physics (IOMP) World Congress at Nice, the formation of SEAFOMP was endorsed by member countries. The South East Asian Federation of Organizations for Medical Physics (SEAFOMP) was officially accepted as a regional chapter of the IOMP at the Chicago World Congress in 2000 with five member countries, viz. Indonesia, Malaysia, Philippines, Singapore and Thailand. Figure 2 shows the logo of SEAFOMP. At that time, the founding members of SEAFOMP were Anchali Krisanachinda and Ratana Pirabul from Thailand, Kwan-Hoong Ng from Malaysia, Agnette Peralta from the Philippines, Djarwani S Soejoko from Indonesia and Toh-Jui Wong from Singapore (Figure 3). Prof. Kwan-Hoong Ng served as the founding president until 2006. Two other countries joined subsequently: Brunei (2002) and Vietnam (2005).

The objectives of SEAFOMP are to promote (i) co-operation and communication between medical physics organizations in the region; (ii) medical physics and related activities in the region; (iii) the advancement in status and standard of practice of the medical physics profession; (iv) to organize and/or sponsor international and regional conferences, meetings or courses; (v) to collaborate or affiliate with other scientific organizations. SEAFOMP has a complementary and synergistic relationship with AFOMP in moving medical physics forward in the region.

Since the formation of SEAFOMP, it has been organizing a series of congresses to promote scientific exchange and mutual support. The South East Asian Congress of Medical Physics (SEACOMP) series were held respectively in Kuala Lumpur (2001), Bangkok (2003), Kuala Lumpur (2004) and Jakarta (2006).

### 1^st^ SEACOMP (Kuala Lumpur 23 - 24 April 2001)

**Figure 4:**
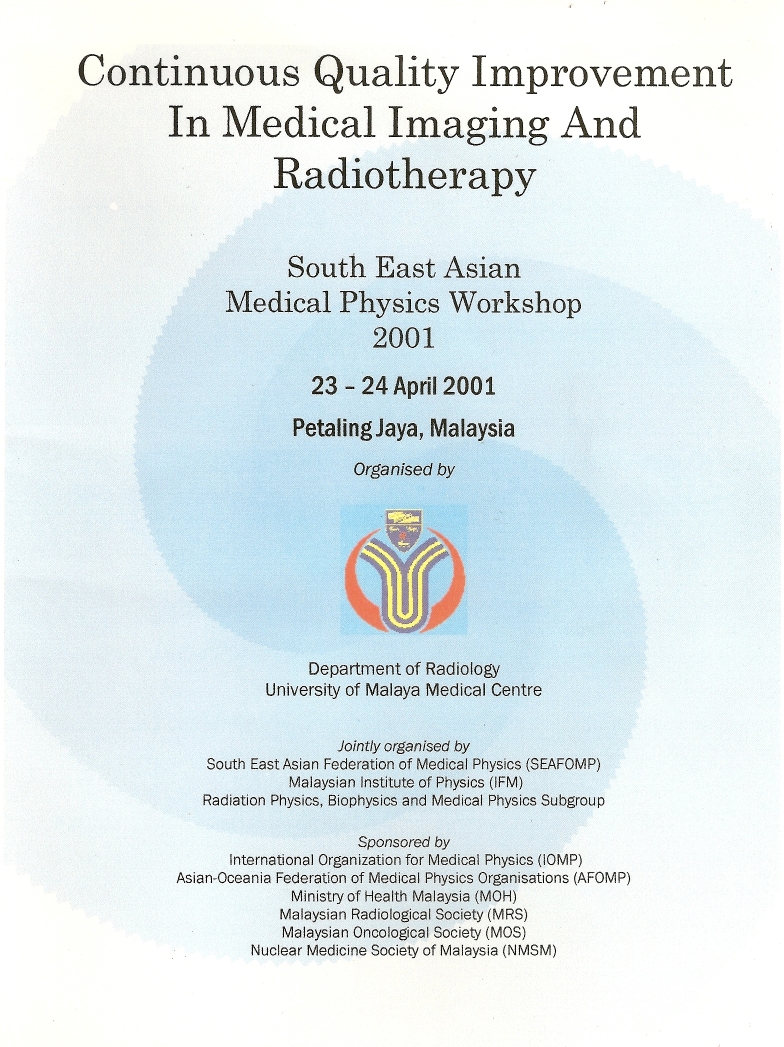
Program book cover for 1st SEACOMP.

The South East Asian Medical Physics Workshop 2001 with the theme of ‘Continuous Quality Improvement in Medical Imaging and Radiation Therapy’ was organized by the Department of Radiology, University of Malaya Medical Centre (UMMC) and jointly organized by SEAFOMP, IFM (Radiation Physics, Biophysics and Medical Physics Subgroup). It was sponsored by IOMP, Asia-Oceania Federation of Organizations for Medical Physics (AFOMP), Ministry of Health Malaysia, Malaysian Radiological Society, Malaysian Oncological Society and Nuclear Medicine Society of Malaysia. It was retrospectively renamed as the First SEACOMP. Figure 4 shows the program book cover for the 1^st^ SEACOMP.

The Workshop was directed by Prof. Gary Fullerton, Radiological Sciences Division, University of Texas Health Science Center, San Antonio, USA and Associate Prof. Kwan-Hoong Ng, Department of Radiology, University of Malaya Medical Centre, Kuala Lumpur, Malaysia.

The invited faculty include: Prof. Gary Fullerton (USA), Ing. Geert Carrein (Belgium), Assoc. Prof. BJJ Abdullah (Malaysia), Assoc. Prof. Sazilah Ahmad Sarji (Malaysia), Assoc. Prof. Ahmad Zakaria (Malaysia), Assoc. Prof. Kwan-Hoong Ng (Malaysia), Assoc. Prof Hang-See Ong (Malaysia), Dr. Matin Mellor Abdullah (Malaysia), Mr. Hwee-Beng Wang (Malaysia), Mr. Taiman Kadni (Malaysia), Dr. James Lee Cheow Lei (Singapore), Dr. Anchali Krisanachinda (Thailand).

This course was a follow-up of a very successful IOMP-endorsed Medical Physics Workshop led by Prof. Larry DeWerd held during 10-11 April 2000. The current course aims at medical physicists, radiographers, radiologists, radiation oncologists, biomedical engineers and allied health professionals from South East Asia and other countries. It objective is to address quality issues in medical imaging and radiation therapy – quality assurance, protocols, quality training and professional development, and emerging technologies. Some emphasis was focused on magnetic resonance imaging, digital imaging, balancing image quality and radiation dose, nuclear medicine, and precision radiotherapy.

About 110 delegates attended, representing 17 countries: Belgium, Brunei, Canada, Germany, India, Indonesia, Libya, Malaysia, Mongolia, Myanmar, Pakistan, Philippines, Singapore, Taiwan, Thailand, USA, and Vietnam. The workshop attracted multi-disciplinary participation from medical physicists, radiographers, radiologists, radiation oncologists, biomedical engineers, equipment vendors, technicians, students, administrators, regulators, etc. The course also incorporates a scientific poster session for participants to present their works. Awards for best poster presentations were given. A mini trade exhibition was also held.

### 2^nd^ SEACOMP (Bangkok 12-14 November 2003)

**Figure 5:**
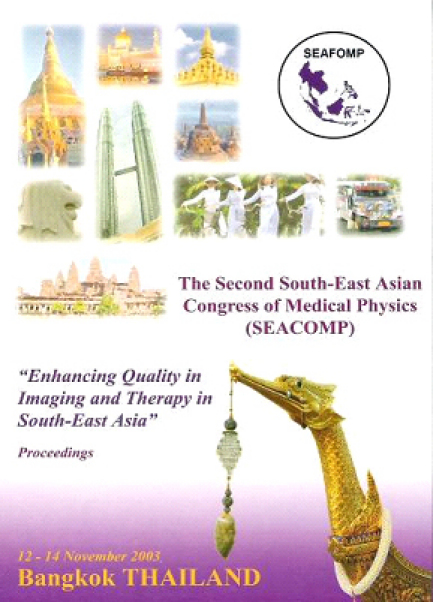
Program book cover for 2nd SEACOMP.

The theme of this congress is “Enhancing quality in imaging and therapy in South-east Asia". This congress was directed by Dr. Anchali Krisanachinda and organized by the Thai Medical Physicists Society and the Faculty of Medicine, Chulalongkorn University. The congress was co-sponsored by IOMP and the Institute of Physics and Engineering in Medicine (IPEM). It also received the endorsement and support from AFOMP and European Federation of Organizations in Medical Physics (EFOMP). The IPEM sponsored travel grant for two eminent speakers, namely Dr. Paul Shrimpton from the National Radiological Protection Board (NRPB) and Dr. JA Evans from the University of Leeds. Figure 5 shows the program book cover for 2^nd^ SEACOMP.

The congress comprises of two plenary lectures, one keynote lecture, one refresher course, 14 invited talks, seven proffered papers and three posters. A new feature of the congress is the seven QA workshops on computed tomography, stereotactic radiosurgery, ultrasonography, single photon emission computed tomography, IMRT, mammography and brachytherapy. These workshops encompass hands-on and demonstration sessions.

The two plenary lectures delivered were: “Status of Medical Physicist in Thailand” by Prof. Makumkrong Poshyachinda and “Patient Protection for Diagnostic Radiology in the 21^st^ Century” by Dr. Paul Shrimpton. The other invited speakers include Dr. JA Evans, Mr. Ahmad Shariff Hambali, Dr. Peter Homolka, Prof. Anchali Krisanachinda, Mr. Louis Lee, Prof. Franco Milano, Dr. RM Millar, Prof. Kwan-Hoong Ng, Ms. Agnette Paralta, Dr. Frantisek Pernicka, Dr. Madan Rehani, Prof. Djarwani Soejoko, Mr. Hwee-Beng Wang, and Dr. Shuji Yamamoto.

About 150 participants attended the congress, representing some 16 countries: Australia, Austria, Czech Republic, Hong Kong, China, India, Indonesia, Italy, Japan, Malaysia, Nepal, Philippines, Singapore, Thailand, United Kingdom and USA.

### 3^rd^ SEACOMP (Kuala Lumpur 27 - 29 September 2004)

**Figure 6:**
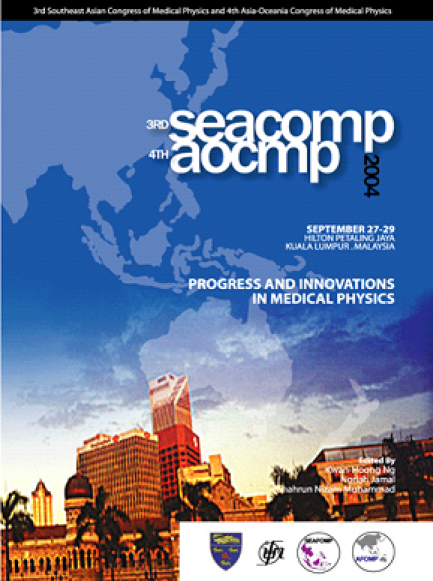
Program book cover for 3rd SEACOMP.

The 3^rd^ SEACOMP was also the 4^th^ Asia-Oceania Congress of Medical Physics (AOCMP). The organizing chairman was Prof. Kwan-Hoong Ng. It was jointly organized by the Department of Radiology, University of Malaya, Malaysia, Radiation Physics, Biophysics and Medical Physics Subgroup of the Malaysian Institute of Physics (IFM), SEAFOMP and AFOMP. It was supported by the IOMP and Abdus Salam International Centre for Theoretical Physics (ICTP). It was also endorsed by the International Union for Physical and Engineering Sciences in Medicine (IUPESM), EFOMP, American Association of Physicists in Medicine (AAPM), IPEM, College of Radiology, Academy of Medicine Malaysia (CoR), and the Ministry of Health Malaysia. Figure 6 shows the program book cover for 3^rd^ SEACOMP.

About 220 participants attended the congress, representing some 23 countries: Australia, Austria, Belgium, Brunei, China, Germany, Hong Kong China, India, Indonesia, Ireland, Italy, Japan, Korea, Malaysia, Myanmar, Netherlands, Philippines, Singapore, Sweden, Thailand, United Kingdom, USA and Vietnam.

A most appropriate theme “Progress and Innovations in Medical Physics” was chosen to reflect the status and direction of medical physics in this fast-growing region. The high scientific standard has been reflected by the 11 plenary addresses, 16 keynote lectures, 4 refresher courses, 1 symposium, 22 proffered papers and 28 posters. The topics covered include: biophysics, computing, digital imaging, dosimetry, education in medical physics, functional imaging, image processing, biomedical instrumentation, magnetic resonance imaging, medical electronics, medical informatics, modeling, molecular imaging, non-ionizing radiation, nuclear medicine, physics of human body, quality assurance, radiation protection, radiation therapy, radiobiology, radiological physics, radiology, regulations and organizations, signal processing, and ultrasound. A trade exhibition was also held.

**Figure 7:**
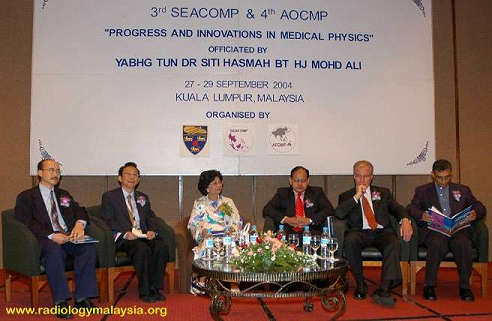
The opening ceremony of the 3rd SEACOMP was graced by YA Bhg Tun Dr. Siti Hasmah, the former first lady of Malaysia.

The congress began with an Opening Ceremony graced by YA Bhg Tun Dr Siti Hasmah bt Hj Mohd Ali (former First Lady of Malaysia who was a medical doctor). Tun Dr Siti delivered her keynote address on “Medical Physics in Women’s Health” (Figure 7) [2]. She spoke on the role of medical physicists in cooperation with other medical professionals, biomedical engineers and scientists in contributing to the prevention, diagnosis and treatment of diseases.

In honour of the late Prof. John Cameron who has contributed tremendously to the development of medical physics in this region, an eponymous lecture was set up. The inaugural John Cameron Lecture was delivered by Prof. Willi Kalender from Germany. His lecture was titled “Recent Developments in Volume CT Scanning”. Subsequently the audience viewed a recorded lecture by Prof. John Cameron on “Imagination and Creativity in Medical Physics” and had a teleconferencing session with him.

Other invited speakers include Prof. Barry J Allen (St George Hospital, Australia), Prof. Shanglian Bao (Peking University, China), Prof. Alun Beddoe (University of Birmingham, UK), Prof. John Cameron (University of Wisconsin, USA), Dr. KY Cheung (Prince of Wales Hospital, Hong Kong, China), Dr. Rehir Dahalan (AELB, Malaysia), Dr. David J Dowsett (Dublin, Ireland), Dr. JA Evans (University of Leeds, UK), Prof. Kiyonari Inamura (Kansai University, Japan), Mr. Taiman Kadni (MINT, Malaysia), Prof. Willi Kalender (Institut fuer Medizinische Physik, Germany), Prof. Anchali Krisanachinda (Chulalongkorn University, Thailand), Prof. Franco Milano (Università di Firenze, Italy), Prof. Kwan-Hoong Ng (University of Malaya, Malaysia), Ms. Agnette Peralta (Manila, Philippines), Prof. Djarwani Soejoko (Jakarta, Indonesia), Prof. Tae Suk Suh (Catholic University, Korea), Dr. Shuji Yamamoto (Osaka University, Japan), and Dr Allan Wilkinson (Cleveland Clinic Foundation, USA).

SEACOMP has initiated the tradition of awarding the best student presentation awards and this has stimulated much interest among the students. The students were given awards for best student presentations, both oral and poster, to encourage excellence in this field. Book prizes were generously donated by Medical Physics Publishing.

The abstracts were published in the Australasian Physical & Engineering Sciences in Medicine Vol. 27 No. 2 June 2004 issue. A 380-page proceedings, in hard and soft copies, were also published and distributed to all the participants.

### 4^th^ SEACOMP (Jakarta 7 - 11 November 2006)

**Figure 8:**
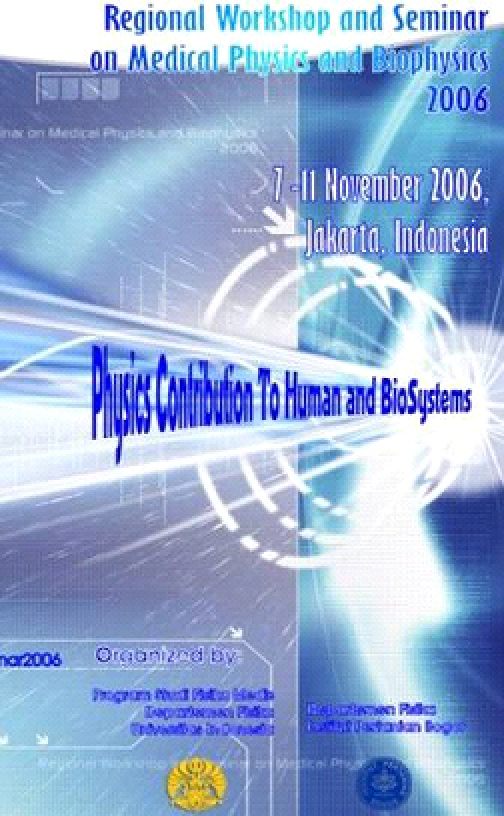
Program book cover for 4th SEACOMP.

The 4^th^ SEACOMP, with the theme ‘Physics Contribution to Human and Biosystem’ was jointly organized by the Department of Physics, University of Indonesia; Department of Physics, Bogor Institute of Agriculture; Indonesian Medical Physics and Biophysics Association. The local organizers were headed by Dr. Rachmat Adi and Prof. Djarwani Soejoko. Figure 8 shows the program book cover for 4^th^ SEACOMP.

The invited international speakers include: Prof. Kwan-Hoong Ng, Dr. KY Cheung (Prince of Wales Hospital, Hong Kong, China), Prof. Anchali Krisanachinda (Chulalongkorn University, Thailand), Dr. Karlis Gross (Biomaterials Engineering, University of Melbourne), Dr. Martin Law (Queen Mary Hospital, Hong Kong), and Mr. TJ Wong (National Cancer Center, Singapore). The local invited speakers include regulators from the National Atomic Energy Agency and the National Nuclear Energy Control Board, radiologist and radiation oncologist.

About 126 participants from 7 countries attended the congress: Australia, Hong Kong China, Indonesia, Malaysia, Philippines, Singapore, Sweden and Thailand. The congress consisted of 3 plenary lectures, 10 keynote addresses, 1 symposium, 26 proffered papers and 5 poster presentations.

A radiation dosimetry workshop was held from 9-11 Nov. This workshop consisted of lectures and laboratory work, and was attended by physicists from several hospitals in Indonesia and staff from the Ministry of Health. The trainers in the lectures were Dr. James Lee (National Cancer Center, Singapore), Mr. Mohd. Izhwan Goh (National University Hospital, Singapore), Dr. KY Cheung (Prince of Wales Hospital, Hong Kong), and Prof. Djarwani S Soejoko (Department. of Physics, University of Indonesia).

The laboratory work was held at Radiotherapy Department, Pertamina Hospital Center and they also visited the Radiotherapy Department, Dr. Cipto Mangunkusumo Hospital Center. The local trainers were: Dwi Seno K Sihono (Department. of Physics, University of Indonesia) and Indra Johannes (Radiotherapy Department, Pertamina Hospital Center).

We continued with the SEACOMP tradition to grant meritorious awards to outstanding oral and poster presentation for students. These awards were created to encourage students to embark on research and to promote the profession of medical physics. The judging committee headed by both Dr. KY Cheung and Mr. TJ Wong remarked that the overall quality was excellent and a total of eight awards was given out to the following students: Imada Fatma, Indra Yohannes (Indonesia); Nantaporn Naiyanet, Navapan Pitaxtarnin (Thailand); Mohamad Fahdillah, Chai-Hong Yeong, Hwee-Shin Soh, Saidatul Ardeenawatie (Malaysia).

### 5^th^ SEACOMP (Manila 21 -23 November 2007)

**Figure 9:**
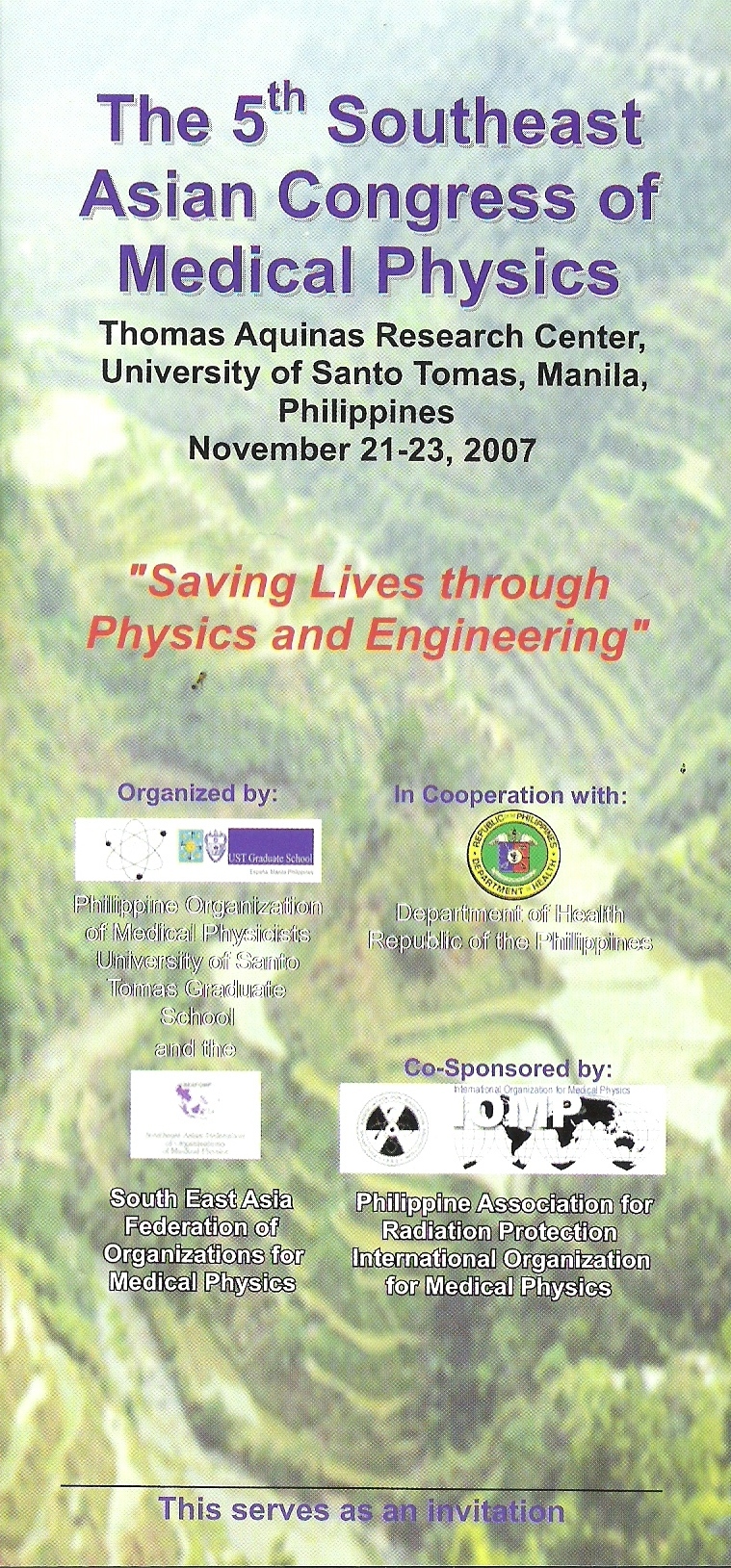
Brochure for 5th SEACOMP.

The 5^th^ SEACOMP, with the theme “Saving Lives Through Physics and Engineering” was organized by the Philippine Organization of Medical Physicists (POMP) and the UST Graduate School. It is supported by the IOMP Education and Training Committee, the Department of Health, the Department of Science and Technology, and the University of Santo Tomas. The local organizing team was led by Ms. Agnette Peralta and Prof. Lilian Sison. Figure 9 shows the brochure design for 5^th^ SEACOMP.

There were 124 participants from 11 countries: Australia, Brunei, Hong Kong, Italy, Japan, Korea, Philippines, Malaysia, Saudi Arabia, Sweden and Thailand. The scientific program consists of 36 oral presentations, 6 posters and 16 lectures. In addition, there were 3 refresher courses on “QA with Treatment Planning Systems”, “QA with Mammographic Systems”, and “QA with PET-CT Systems” and also a symposium on “Education and Training of Medical Physicists” where Malaysia, Thailand and the Philippines shared and learned from each other’s experiences. A post –conference refresher course on “QA with Linacs” was also held for the local participants..

The highlight of the Congress was the 2^nd^ John Cameron Memorial Lecture which was delivered by Professor Kwan-Hoong Ng of the University of Malaya, who is also the founding president of SEAFOMP and president-elect of the Asia-Oceania Federation of Organizations for Medical Physics (AFOMP). Prof. Ng addressed the relevancy of medical physics in his lecture, aptly titled “Medical Physics in 2020: Will We Still Be Relevant?”

Other invited speakers were IOMP President Prof. Barry Allen, AFOMP President Prof. Kiyori Inamura, former AFOMP President Dr. KY Cheung, IAEA expert, Dr. John Drew, Dr. Somsak Wanwilairat of Chiang Mai University, Dr. David Causer of the Royal Perth Hospital, Prof. Franco Milano of the University of Florence, and SEAFOMP President Prof. Anchali Krisanachinda. IAEA expert Dr. John Drew also gave a talk on the “IAEA Regional Cooperation Agreement Clinical Training Modules in Radiation Oncology Medical Physics (ROMP)” while Prof. Krisanachinda gave a talk on “The Experience in Piloting the ROMP Clinical Training Modules in Thailand.”

The 5th SEACOMP drew to a close with the honouring of the tradition of SEACOMP and in the spirit of nurturing young aspiring scientists and physicists. Prizes were awarded to the six best student papers upon the recommendation of the Board of Judges headed by Prof. Milano. The awards were given to Mah Yik Hoay and Wan Hazlinda of Malaysia, Thunyarat Chusin and Isra Israngkul Na Ayuthaya of Thailand, and Darrin Casipong and Hazel Faustino of the Philippines.

## SEAFOMP – THE WAY FORWARD

Since the official founding in 2000, SEAFOMP has grown in strength and stature. Medical physics is experiencing rapid growth and we have witnessed the rapid deployment of PET/CT, IMRT, tomotherapy, digital radiology, functional MRI, PACS, and teleradiology in Southeast Asia and the Asia-Pacific region. In order to utilize these modalities optimally and safely we need to keep abreast and be educated and innovative. Indeed, medical physics is well and alive in Southeast Asia, and we hope that it will continue to do so under the driving force of SEAFOMP [3].

The executive committee of SEAFOMP (2000-2006)

- President: Prof. Kwan Hoong Ng, Malaysia
- Vice President: Dr Anchali Krisanachinda, Thailand
- Secretary: Ms Agnette Peralta, Philippines
- Treasurer: Mr Toh Jui Wong, Singapore
- Auditor: Dr Djarwani Soejoko, Indonesia

The executive committee of SEAFOMP (2007-2009)

- President: Prof. Anchali Krisanachida, Thailand
- Vice President: Ms Agnette Peralta, Philippines
- Immediate Past President: Prof. Kwan-Hoong Ng, Malaysia
- Secretary: Dr James Lee, Singapore
- Treasurer: Dr Rachmat Widodo Adi, Indonesia

We will continue to witness the growth and progress of medical physics through the 5th SEACOMP which is going to be held in Manila, Philippines on 21–23 November 2007 and the 6th SEACOMP to be held in Vietnam (2008).
